# Studying Hallucinations Within the NIMH RDoC Framework

**DOI:** 10.1093/schbul/sbu011

**Published:** 2014-05-21

**Authors:** Judith M. Ford, Sarah E. Morris, Ralph E. Hoffman, Iris Sommer, Flavie Waters, Simon McCarthy-Jones, Robert J. Thoma, Jessica A. Turner, Sarah K. Keedy, Johanna C. Badcock, Bruce N. Cuthbert

**Affiliations:** ^1^San Francisco VA Medical Center, San Francisco, CA;; ^2^Department of Psychiatry, University of California, San Francisco, CA;; ^3^Division of Adult Translational Research, National Institute of Mental Health, Bethesda, MD;; ^4^Department of Psychiatry, Yale-New Haven Psychiatric Hospital, New Haven, CT;; ^5^Psychiatry Department, University Medical Center, Utrecht, Netherlands;; ^6^Centre for Clinical Research in Neuropsychiatry, School of Psychiatry and Clinical Neuroscience, The University of Western Australia, Perth, Australia;; ^7^Graylands Hospital, North Metro Health Service Mental Health, Perth, Western Australia;; ^8^ARC Centre for Excellence in Cognition and Its Disorders, Department of Cognitive Science, Macquarie University, Sydney, Australia;; ^9^Department of Psychology, Durham University, Durham, UK;; ^10^Department of Psychiatry, University of New Mexico, Albuquerque, NM;; ^11^Psychology Department and Neuroscience Institute, Georgia State University, Atlanta, GA;; ^12^Department of Psychiatry and Behavioral Neuroscience, University of Chicago, Chicago, IL;; ^13^School of Psychology, University of Western Australia, Crawley, Western Australia;; ^14^Clinical Research Centre, North Metropolitan Health Service - Mental Health, Mount Claremont, Western Australia

**Keywords:** hallucinations, Research, Domain, Criteria, RDoC

## Abstract

We explore how hallucinations might be studied within the National Institute of Mental Health (NIMH) Research Domain Criteria (RDoC) framework, which asks investigators to step back from diagnoses based on symptoms and focus on basic dimensions of functioning. We start with a description of the objectives of the RDoC project and its domains and constructs. Because the RDoC initiative asks investigators to study phenomena across the wellness spectrum and different diagnoses, we address whether hallucinations experienced in nonclinical populations are the same as those experienced by people with psychotic diagnoses, and whether hallucinations studied in one clinical group can inform our understanding of the same phenomenon in another. We then discuss the phenomenology of hallucinations and how different RDoC domains might be relevant to their study. We end with a discussion of various challenges and potential next steps to advance the application of the RDoC approach to this area of research.

In this article, we explore how hallucinations, a cardinal symptom of schizophrenia spectrum disorders that occur frequently in other psychiatric and neurologic conditions, might be studied within the NIMH Research Domain Criteria (RDoC) framework. The RDoC initiative asks investigators to step back from diagnoses based on heterogeneous clusters of symptoms and, instead, to focus on basic dimensions of functioning across the wellness spectrum that might relate to various aspects of symptoms. Inverting the usual paradigm of starting with symptoms and seeking a corresponding pathophysiology is one divergence from traditional approaches. A second divergence is that while RDoC is very much directed toward an understanding (and eventual remediation) of symptoms, a symptom in this approach is better understood as “an abnormality of some degree that can be expressed quantitatively with respect to its deviation from the usual operation of the function(s) attributed to the construct.^1^” In other words, traditional subjective notions of symptoms are supplanted by an emphasis on quantification and relationships to neural systems.

We start with a description of the objectives of the RDoC initiative and its domains and constructs. This is followed by a summary of clinical and nonclinical populations who experience hallucinations (described in more detail in this issue, Johns et al^2^), as we ask whether hallucinations studied in one patient group can inform our understanding of the same phenomenon in another. We then discuss the phenomenology of hallucinations and how various RDoC domains might be relevant to their study. We end with a discussion of various challenges and potential next steps to advance the application of the RDoC approach to this area of research.

For this heuristic exercise, we focus on auditory hallucinations (AHs), reflecting current societal and research interest. Because the experience is predominantly verbal, we narrow our focus to auditory verbal hallucinations. With minor adjustments, the same framework can be applied to hallucinations in other sensory modalities and to AHs that are nonverbal (an in-depth discussion of visual hallucinations appears in this issue, Waters et al^3^).

## The RDoC Initiative

### Objectives

One objective in NIMH’s strategic plan is to develop, for research purposes, new ways of classifying mental disorders based on dimensions of observable behavior and neurobiological measures. This stems from dissatisfaction with the slow progress the field has made in translating advances in neuroscience into improved treatments for mental disorders, which is due in part to constraints imposed by diagnostic systems used in psychiatry research. Indeed, one reason for poor efficacy of psychiatric drugs may be “the artificial grouping of heterogeneous syndromes with different pathophysiological mechanisms into one disorder.^4^” Current approaches for diagnosing and classifying participants precede modern neuroscience and have set investigators off to hunt for genes and pathophysiology to explain overlapping and heterogeneous clusters of symptoms. This has yielded few breakthroughs in understanding and treating mental disorders.

The mandate for RDoC is to consider psychopathology in terms of maladaptive extremes along a continuum of normal functioning, to promote a translational emphasis. Rather than starting with a cluster of symptoms defined as a “diagnosis” by the Diagnostic and Statistical Manual of Mental Disorders (DSM) or the International Classification of Diseases (ICD) and then trying to establish associated pathophysiology, the RDoC approach encourages us to start with what is known about healthy, adaptive behavioral and neural circuit functioning, and then to understand how alterations in these systems could eventuate in various types of symptoms and impairments. With its focus on dimensions of functioning and behavioral signs rather than diagnosis, RDoC aims to overcome limitations of diagnostic systems for mental disorders (a similar cognitive neuropsychiatric approach was taken earlier by others including seminal contributions from Frith,^5^ David,^6^ and Halligan and David^7^).

### Domains and Constructs

How do we approach studying “hallucinations” from the RDoC principle of starting from normative experience, as hallucinations are typically, but not always,^8^ considered an abnormal experience? We assume these experiences result from alterations in functioning of normal brain circuits, which have evolved to adaptively serve many complex human behaviors and processes. RDoC has organized these neurobehavioral processes into 5 domains: cognitive systems, negative valence systems, positive valence systems, systems for social processes, and arousal/regulatory systems. Within each domain there is a set of related constructs, defined at a series of NIMH workshops, which can be studied using various “Units of Analysis” (from genes to self-report). This multidimensional approach is intended to facilitate integration of basic research in genetics, molecular and cognitive neuroscience, physiology, and behavior, and accelerate translation of this knowledge into research focused on well-defined clinical problems. Rather than being reductionistic, the goal is to include multiple units of analysis in an integrative approach. A segment of the RDoC matrix appears with one exemplar construct in table 1 and another in table 2. The units of analysis comprise columns. Construct names are in bold, and definitions are provided in footnotes below. The complete matrix and definitions for all constructs, as well as citations for publications that elaborate the RDoC framework, is here http://www.nimh.nih.gov/research-priorities/rdoc/index.shtml.

**Table 1.** Auditory Perception Construct

Genes	Molecules	Cells	Circuits	Physiology	Behavior	Self-Reports
Brain-derived neurotrophic factor	Glutamate; gamma- aminobutyric acid; *N*-methyl- d-aspartate; serotonin; acetylcholine	Cochlear hair cells; ribbon synapses; cortical and limbic inhibitory interneurons	Nodes in circuits; cochlea; brainstem; medial geniculate nucleus; primary auditory cortex; superior temporal gyrus; anterior insula; inferior colliculus. Circuits: dorsal/ ventral streams; corticofugal system	Sensory event- related potentials (eg, P50, N1); auditory steady- state response; Intracortical EEG; mismatch negativity; P3a; startle and prepulse inhibition; neural oscillations (eg, gamma- band response); adaptation/ habituation; regulation of hemodynamic components of sensory response and habituation	Stimulus detection;spatial localization; perceptual identification, priming, and learning	Auditory hallucinations; hyperacusis

*Note*: Paradigms—tone matching; deviance detection, regularity and change detection; McGurk (multisensory); auditory scene perception; bistability; novelty/oddball detection; speech in noise; cross-modal interactions; auditory masking; manipulation of interstimulus interval and intensity; object perception; categorization; gating; same-different tasks; tone detection (eg, just-noticeable-difference tasks), action-perception loops.

**Table 2.** Acute Threat Construct

Genes	Molecules	Cells	Circuits	Physiology	Behavior	Self-Reports
BDNF; 5-hydroxytryptamine receptor; corticotropin- releasing hormone gene; GABA receptors; glutamate system; NMDAR; opioid system; COMT; cannabinoid system; dopamine active transporter; CaM, MAP and PI-3 kinase; PK-A; PK-C; acetylcholine; norepinephrine; stathmin; TRBC5	NMDAR; glutamate; dopamine; serotonin, BDNF; GABA; cortisol/ corticosterone; endogenous cannabinoids; orexin; neuropeptide Y; corticotropin releasing factor family; fibro blast growth factor 2; oxytocin; vasopressin; cholecystokinin, neuropeptide S; neurosteroids	Neurons; glia; pyramidal cells; GA BAergic cells	Central nucleus; basal and lateral amygdala; Periaqueductal gray; ventral/ posterior hippocampus; dorsal/anterior hippocampus; lateral PFC/ insula; ventromedial PFC; dorsomedial PFC; orbitofrontal cortex; hypothalamus; dorsal ACC; rostral/ventral ACC; medial amygdala; pons; autonomic nervous system; locus coeruleus	Fear potentiated startle; context startle; skin conductance; heart rate; blood pressure; eye tracking; facial electrom yography; respiration, pupillometry	Freezing; response time; avoidance; response inhibition; social approach; analgesia; approach (early development); risk assessment; facial expressions	Fear survey schedule; Beck Anxiety Inventory; State- Trait Anxiety Inventory; Subjective units of distress; Fear Questionnaire; Trait Fear Inventory; Eilam ethogram; Albany Panic and Phobia Questionnaire

*Note*: Paradigms—fear conditioning; viewing aversive pictures or films; emotional imagery; open field test. BDNF, brain-derived neurotrophic factor; GABA, gamma-aminobutyric acid; NMDAR, *N*-methyl-d-aspartate receptor; COMT, catechol-*O*-methyltransferase; CaM, calmodulin-dependent; MAP, mitogen-activated protein; PI-3, phosphoinositide-3; PK, protein kinase; TRBC5, short transient receptor potential channel 5; PFC, prefrontal cortex; ACC, anterior cingulate cortex.

In this step of our exercise, we must identify which circuit-based constructs might be involved in the experience of hallucinations (while RDoC is a research framework intended to accommodate additional dimensions, for purposes of this discussion we consider only the extant constructs). First, we need to consider the wide variety of experiences people report in describing their AHs and evaluate whether each variant is linked to a specific RDoC domain, whether all variants result from a single domain, or whether there are both shared and unique domains.

## Who Hears Voices? Commonalities and Differences in AH Across Nonclinical and Psychiatric Groups

RDoC’s dimensional approach encourages investigators to think beyond between-group, patients-vs-controls research designs and, instead, to design studies that allow analysis of the full range of a dimension of interest, including clinical as well as nonclinical groups and remaining agnostic with regard to diagnosis. AHs are fertile ground for this approach. While most empirical attention has been focused on people diagnosed with schizophrenia (~70% of whom experience AHs), AHs also occur in a wide range of other disorders and in the nonclinical population.^2,9–11^

### AHs Across Diagnoses

Consistent with RDoC, our understanding of AHs might be enriched by moving beyond the confines of categorical diagnoses and incorporating what is known about AHs in other populations/diagnoses. AHs are reported in borderline personality disorder, post-traumatic stress disorder, temporal lobe epilepsy, traumatic brain injury, social trauma, sexual or physical abuse, and acquired deafness, to name a few. While most studies of AHs focus on AVH, we might also ask about related phenomena such as tinnitus.

Within the RDoC framework, we can address a primary question of “equifinality^12^”: If the symptom is the same in 2 different clinical groups (eg, an angry voice commenting negatively), but the diagnosis (alcoholism vs posttraumatic stress disorder) and etiologies (alcohol intoxication vs childhood adversity) are different, can we assume the same mechanisms are involved^13^? If so, we can use AHs with well-known origins as a model for AHs in other psychiatric disorders that are not clearly understood.

### AHs in the Nonclinical Population

As described in more detail in this issue,^2^ in recent years there has been increasing focus on voice hearing in the nonclinical population. Here it is useful to distinguish between people in the general population who have brief and rare voice-hearing experiences (such as hearing one’s name called out) and people who have more frequent and extended voice-hearing experiences, which may more closely approximate the typical form of clinical experiences. After excluding people in the general population who hear voices and do in fact meet criteria for a psychotic disorder, approximately 0.2%/year of the general population hear voices saying “quite a few words or sentences”.^14^ It is such individuals that we refer to here as nonclinical hallucinators.

There is considerable debate regarding whether voices experienced by nonclinical voice hearers and psychotic individuals are on a continuum, with the pros and cons discussed in Johns et al^2^. An important distinction here is that psychotic individuals more often report negative voices that they cannot control, resulting in increased distress^15^—suggesting the RDoC negative valence system domain may be useful in differentiating between psychotic and nonclinical voice hearers.

## What Is It Like to Hear Voices?

To begin to dissect hallucinations along RDoC lines, we need to understand the phenomenon, which is described across cultural, social, and historical contexts in this issue.^16^ While speaking specifically about auditory verbal hallucinations (AVH), Jones^17^ summarizes the breadth and relevance of phenomenological heterogeneity relevant to our focus on AHs:

“The term AVH encapsulates a diverse phenomenological experience, which may involve single and/or multiple voices, who may be known and/or unknown, speaking sequentially and/or simultaneously, in the first, second, and/or third person and which may give commands, comments, insults, or encouragement. Given the prima facie heterogeneity of AVHs, it is surprising that only recently has the suggestion been made that ‘‘perhaps we now have to consider further subcategorisations of [auditory] hallucinations’’ (David, 2004, p. 118^18^) and that this is likely to have important clinical, theoretical, and empirical implications.”

Accepting the premise that various aspects of normative cognition that give rise to diverse aspects of inner experience might also result in distinct features of AHs,^19^ we review various aspects of experiences people report when describing their AHs and attempt to relate them to RDoC constructs. Throughout, we attempt to identify constructs that link closely to existing research on AHs and provide illustrative examples of research using RDoC principles.

**Fig. 1.**
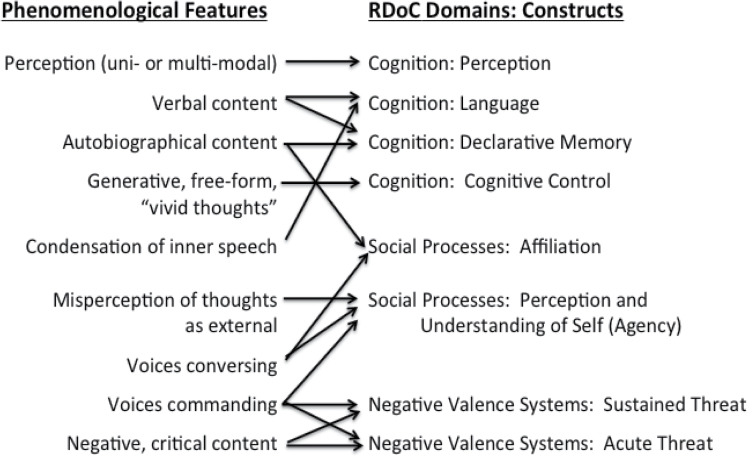
Possible relationships between hallucinatory phenomenology and RDoC constructs. Note: Some aspects of hallucinatory phenomenology might be adequately characterized within one construct and others might reflect the interactive contributions of multiple constructs. RDoC, Research Domain Criteria.

No single RDoC construct captures the complex phenomenology of AHs. Instead, the phenomenology and various models of AHs place the study of AHs at the intersection of several of the RDoC domains. Although the structure of the RDoC matrix suggests boundaries among constructs, given the densely integrated and interconnected nature of the brain’s circuits, it is understood that constructs function interactively. The most promising empirical approaches to hallucinations will likely involve examining intersection among constructs. This is illustrated in figure 1.

### Are Hallucinations Different From Normal Perception?

The colloquial term used to refer to AHs, namely “hearing voices,” suggests that auditory perception systems will by definition be involved in AHs. However, although the majority who endorse AHs describe them as a perceptual experience, others report their AHs are more like their own thoughts than perceptions of voices.^20^ Some report a sense of otherness or the presence of another person. Less often, they may describe an experience of “pure meaning” being communicated.^21^

Nevertheless, for most hallucinatory phenomenology, there is a straightforward link to the RDoC Perception construct. This construct is part of the cognition domain and includes 3 subconstructs: auditory perception, visual perception, and olfactory/somatosensory/multimodal perception. The construct captures not only perceptual thresholds and acuity, but the totality of the perceptual system including integration of percepts and cross-modal integration.

Aleman and Larøi^22^ define hallucinations as “a conscious sensory experience that occurs in the absence of corresponding external stimulation of the relevant sense organ and has sufficient sense of reality to resemble a veridical perception.” This suggests that they are like normal perception, with all the complexities and contextual dependencies associated with perception that can shape the content of AHs, the personality of voices, and their relationship to the hearer. This is reminiscent of Arieti’s “listening attitude” in describing the origin of hallucinatory phenomena.^23^ Indeed, current models of human voice perception are proving fruitful in guiding our understanding of AHs.^24,25^

Although occasionally voices are reported to sound robotic or be cast in nondescript word images, hallucinatory voices predominantly recreate specific speakers that sound precisely the same from day to day complete with characteristic tonality, accents, etc., and people generally come to recognize acoustic signatures similar to how we can recognize someone’s voice as quickly as we recognize the face. The neurobiology supporting this kind of experience must involve auditory perception systems, and perhaps failures of those systems in individuals reporting AHs.

While some aspects of phenomenology such as perceived reality,^26^ location,^27^ and loudness^28^ relate to activity in, and connectivity between, speech-related areas of the brain, other details of phenomenology (eg, gender of voice) may have less impact on brain activity or connectivity.

### Content and Affect: What Is Being Said

#### Language.

Given that AH content is often, but not always, spoken words, Language is an obvious RDoC construct on which to focus. This construct, from the cognition domain, captures both productive and receptive aspects of language including prosody, phonology, syntax, and semantics related to words, sentences, and discourse. Furthermore, given that some AHs involve atypical processing of inner speech,^29^ and given that reporting of AHs involves comparison of typical and atypical inner experiences,^30^ studying inner speech/language in normally functioning people may help us in our efforts to understand the commonalities between AHs and normal inner speech.

#### Declarative Memory.

A significant proportion of people diagnosed with schizophrenia explicitly report that their voices are either identical or similar to memories of previously heard speech.^19^ As the content of AHs often follows consistent themes and personalities, also implicating retrieval of memories, we need to study brain systems involved in storage and retrieval of Declarative Memory, as well as interactions between those systems and temporal, frontal, and striatal regions. Indeed, there is evidence for cognitive and neurobiological alterations in contextually bound declarative memory in people diagnosed with schizophrenia who experience AHs.^31^ One approach to understanding the role of this construct in AHs might be to study activity in memory-related circuits during AHs in a range of people whose hallucinatory experiences vary according to whether their AHs involve memories. Beside the straightforward hypothesis that declarative memory circuits would activate more for subjects whose AHs involve memories, such a study could also examine activity in areas involved in details of that memory.

#### Negative Valence Systems.

There is little question that AHs described in treatment settings are typically negative, threatening, and derogatory, suggesting involvement of constructs within the negative valence systems domain, including Sustained Threat and Acute Threat. Recent conceptualizations link negative valence in AHs to past trauma and psychosocial stressors, and current distress with the inability to control these experiences in both clinical groups and voice hearers.^32^ Hypervigilance associated with AHs following trauma might best be approached in studies of signal detection failures. Specific negative emotions following trauma, such as shame and guilt/self-blame, may be involved in the generation of the both the experience of, and specifically the negative valence of, AHs.^33^ Indeed, social adversity has been documented as an antecedent condition to AHs.^34^

The idea that memory systems play an important role in hallucinations may help explain the preponderance of negative and uncontrollable AHs. Memories with strong emotional valence, especially those concerning danger and disgust, are most easily triggered, and thus most likely to become conscious when there is disinhibition of the memory retrieval system.^35^ Interactions between the RDoC domains of declarative memory and threat may contribute to AHs as documented by heightened activity in parahippocampal gyrus and amygdala during passive listening of emotional words in patients with schizophrenia who experience AHs.^36^

As noted above, voices heard by nonclinical voice hearers are rarely negatively charged. In fact, negative emotional content of voices may be an important sign that the voice hearer is transitioning toward psychosis.^15^ This type of observation suggests that circuits involved in sustained and acute threats are less engaged when content is positive or neutral than negative and/or distressing.

#### Cognitive Control.

AHs are often described as unintentional and intrusive,^37^ distinguishing them from thoughts that may be more controllable. Cognitive explanations of AHs have thus incorporated the idea that they involve the breakdown in one or more of the executive functions that control and regulate thought and action. Indeed studies show that AHs in psychosis are linked to inhibition failures and difficulties manipulating online information.^38^ This impairment in inhibitory control has been demonstrated along a continuum of AHs in nonclinical and clinical hallucinators.^39^ Sometimes the contents of AHs seem totally nonrepetitive, and as generative and freeform as the speech of another real person, pointing to systems involved in ruminations and failed Cognitive Control. These unbidden voices say things the patient would never say and may be intrusions that co-opt consciousness.

### Source: Who Is Talking?

#### Affiliation and Attachment.

AHs seem to be grounded in our everyday social experiences. For example, their content sometimes reflects the importance of social rank and is a mirror of social relationships in daily life.^40,41^ AHs are typically experienced as issuing from persons, agents, or other beings outside of self. It is the Otherness of experiences that often makes them so compelling. Similarly, there is often a dialogic aspect to the experience, where individuals often describe entering into conversations with voices. The prominent social quality of AHs is underscored by the nonpathological experience of hearing one’s name being called in a crowd or noisy environment.^42^ This common experience appears to happen because hearing our names spoken has great social importance. Another common example is hearing the voice of a lost loved one when grieving.^43^ These are all nonpathological AHs in terms of life interference and base rate, but importantly suggest that an RDoC approach to hallucinations include consideration of social processing systems,^44^ including the Affiliation and Attachment construct.

Social processing systems are also relevant to one explanatory model for hallucinations in psychiatric illnesses, that pre-illness social isolation triggers reorganization of social salience networks, with deleterious top-down effects on sensory processing.^38,45^ Consistent with this illness model is the finding that right temporal activation, which underlies some social aspect of speech perception, precedes and possibly triggers activation in Wernicke’s area itself during AHs.^46^

#### Agency.

One core feature of AHs is that they are experienced as somewhat separate from one’s own mental processes. This lack of subjective experience of self, accompanied by false beliefs that they arise from an external agency, has traditionally been thought to reflect deficits in the sense of agency or self-monitoring. Feinberg^47^ suggested that a basic neural mechanism (efference copy/corollary discharge) might underpin these deficits and ultimately result in AHs. During self-produced actions, corollary discharge signals alert sensory cortex about the expected sensations. If these fail to be effectively transmitted to speech perception regions, thoughts may sound “non-self.” Studies have now found evidence for impaired corollary signals during self-produced speech associated with AHs.^48^ In addition, related approaches such as feed-forward models^49^ and apparent mental causation^50^ have been applied to AHs (see Jones and Fernyhough^51^) as have other fruitful ways forward.^44^ Indeed, clinical and nonclinical groups with AHs have difficulties in identifying their own actions and thoughts, and commonly misattribute self-generated behaviors to an external source.^52^

Importantly, there are several different forms of AHs^19^; some people report “Own Thought” AHs, formerly termed “Gedanken Lautwerden.” The neural mechanisms responsible for self and non-self AHs could be different.^53^ For people who can distinguish between their voices and their responses to the voices, self-monitoring failure is a less parsimonious explanation.

### RDoC and Phenomenology

An important caveat to the preceding discussion is that the initial grouping heuristic of phenomenology may only be a starting point. That is, an RDoC approach would not be confined to selecting individuals with different types of hallucinatory activity and examining differences in measures at the various units of analysis. While this may be a necessary point of departure, it might be seen as a variant of a DSM-based design by identifying levels or types of psychopathology and seeking group differences on biological or behavioral measures. As many have pointed out, overlap between groups in laboratory measures is the norm rather than the exception,^54^ so important heterogeneity will be lost. Rather, a key postulate of RDoC is that it will often be useful to employ a non-symptom measure as the independent variable across the entire range of subjects (eg, neural circuit activity or behavioral performance in laboratory tasks, measured continuously) and examine other measures (including symptoms) as dependent variables. Thus, type and severity of symptoms might be thought of as located probabilistically at the extremes of one or more dimensions in a multidimensional space, such that an understanding of relationships is better achieved by examining the full range of all dimensions rather than starting with a single symptom cluster and seeking its neurobiological correlates. The advantages of such an approach include the potential incorporation of multiple dimensional constructs to converge upon one clinical problem; the perspective of specifying normal distributions against which varying degrees of psychopathology can be referenced; a straightforward pathway to considering developmental trajectories; and an emphasis on quantified tasks based upon specific neural systems. An additional feature of this approach is that it can incorporate data from family members (whether unaffected or symptomatic to varying degrees) and a wide range of healthy controls; such subjects may help define points of nonlinearity beyond which overt psychopathology is more likely or more severe, and thus contribute to an understanding of the precise factors that demarcate the continuity from normal range functioning to various degrees of impairment.

## Next Steps

### Assessment of Phenomenology

Although we argue that work should be informed by the phenomenology we are trying to understand, it is not always easy to get a valid account of hallucinatory experiences in people with a diagnosis of a psychotic disorder, as some may be guarded. However, other people, not seeking clinical care, may be able to give accounts of their experiences that are more helpful in our efforts to understand the relationships between neurobiological activity and the diverse phenomenology of hallucinations (see Johns et al^2^, this issue).

### Drug Effects

Even in a diagnostically homogeneous group, psychotic individuals are typically prescribed a variety of medications that may affect outcomes on research measures. This concern will only be magnified in diagnostically heterogeneous samples that are optimal for RDoC research and will require careful consideration so that medication confounds do not cloud the interpretation of study results. The inclusion of family members and at-risk individuals to be studied along RDoC dimensions can help to sort out these effects. In addition, medication status can help to inform studies using the molecular unit of analysis. For example, individual differences in responsiveness to medication could be the basis for an RDoC study in which hallucinating participants are organized along a dimension according to dopamine receptor occupancy rates following medication administration and then differences in functional activation of specific neural circuits or changes in cognition could be examined using a dimensional analytic approach.^55^

### Associated Psychopathology

It is worth noting that other associated symptoms, most notably delusions, will be similarly challenging to map onto the RDoC matrix due to their complex phenomenology and understudied neuroscience. Nonetheless, the RDoC approach could yield discoveries by examining many of the constructs described above. While delusions often arise to explain the origins of AHs, delusions are usually associated with poor disease insight, which is not always true with AHs. Formal thought disorder is another symptom that is frequently encountered in hallucinating individuals and may involve constructs such as cognitive control. This is most noticeable in schizophrenia patients, but also detectable in nonclinical hallucinators^56^ and even in hallucinating Parkinson patients.^57^ Formal thought disorder also makes it difficult to understand a person’s description of the hallucinatory phenomena.

### Development

Development has been recognized as a critically important aspect of RDoC, akin to an orthogonal third axis in the matrix (although not depicted in the matrix), and is emphasized as an important factor in all domains and their interactions. Future studies of AHs might consider both developmental stages of individuals, informed by our increasing understanding of normal trajectories of neurodevelopment, as well as the “dynamic developmental progression” of hallucinatory experiences.^17^ For example, might functional connectivity between prefrontal areas and amygdala increase in later compared with early illness in individuals who experience increases in the affective content and intensity of AHs over time? (See a discussion of hallucinations in children and adolescents in this issue, Jardri^58^.)

### Hallucinations in Other Modalities

The literature suggests that 50% of people diagnosed with schizophrenia who report AHs also report visual hallucinations.^20,59^ (Visual hallucinations are discussed in this issue, Waters et al^3^). Those reporting AHs are also more likely to report olfactory and tactile hallucinations than those who do not endorse AHs. Visual hallucinations in the absence of AHs are reported much less frequently in people diagnosed with schizophrenia spectrum disorders. The hallucination nidus may start in the voice network but then spread to other modalities. Alternatively, an earlier common path may originate in hippocampus^60^ and connect memories to the language system for AHs and other systems for hallucinations in other modalities.

### Evolution of RDoC Constructs

There are some psychological constructs that have been useful for describing and understanding hallucinations that do not map onto current RDoC constructs. This is not surprising, given how RDoC was developed: The goal of RDoC workshops was to devise a finite set of rigorously validated constructs that could serve both as particularly promising areas for study, and also as exemplars of the new framework for conducting research independent of ICD/DSM disorder categories. A high bar was set for including constructs to avoid weakly validated constructs. However, the list of RDoC constructs will be continually refined and updated in response to new data and investigators are encouraged to examine other promising constructs. While the project is still too new for any data-driven modifications, application of RDoC dimensions to hallucinations will help determine the extent to which combinatorial actions of current functions will account for significant variance, and whether new functions (eg, mirror neuron systems, inner speech, etc.) will add significantly to our understanding.

## Summary

In this article, we explored how AHs might be studied within NIMH’s RDoC framework. RDoC grew out of a need to develop new ways of classifying mental disorders for research purposes, based on dimensions of observable behavior and measurable neurobiology. Although not directly observable or measurable, phenomenology of AHs served as our starting point in considering which RDoC domains are most relevant for study of AHs pathophysiology. The RDoC imperative to study phenomena across the wellness spectrum and without regard for diagnosis challenges us to ask if voices heard by healthy people and people with various diagnoses, including (but not limited to) schizophrenia, are subserved by the same neural mechanisms. The RDoC constructs provide frameworks for asking whether: the phenomenology and neural basis of hallucinated and external sounds and language are the same; memory-related AHs are supported by the same mechanisms supporting normal memory retrieval; AHs of negatively charged voices are supported by neural systems responsible for experiences of sustained and acute threat; AHs of unbidden intrusive thoughts are related to failures of cognitive control; AHs in socially isolated people are supported by social salience networks; and mechanisms underlying the experience of self and non-self voices are the same.

## Funding

National Institute of Mental Health (MH058262 [J.M.F.], MH067073 [R.E.H.], MH073673 [R.E.H.]); Veterans Administration (J.M.F.); VIDI grant from the Netherlands Organization for Scientific Research (I.E.C.S.); K23MH092702 (S.K.K.); National Institutes of Health 2P20GM103472-06 (R.J.T.; J.A.T.); P20RR021938 Phase 1NCRR COBRE (R.J.T.); Wellcome Trust Grant (WT098455 to S.M.-J.); Macquarie University Research Fellowship (S.M.-J.).
